# Effectiveness of the self-regulation educational program in increasing knowledge about COVID-19 in Peruvian soldiers

**DOI:** 10.1590/0034-7167-2022-0498

**Published:** 2023-05-08

**Authors:** Denices Soledad Abarca-Fernández, Jhon Alex Zeladita-Huaman, María Belen Arriaga, Roberto Zegarra-Chapoñán

**Affiliations:** I Universidad Nacional del Altiplano. Puno, Peru; II Universidad Nacional Mayor de San Marcos. Lima, Peru; III Universidade Federal da Bahia. Salvador, Bahia, Brazil; IV Universidad María Auxiliadora. San Juan de Lurigancho, Lima, Peru

**Keywords:** Knowledge, Coronavirus Infections, Military Personnel, Effectiveness, Disease Prevention, Conhecimento, Infecções por Coronavírus, Militares, Efetividade, Prevenção de Doenças, Conocimiento, Infecciones por Coronavirus, Personal Militar, Efectividad, Prevención de Enfermedades

## Abstract

**Objective:**

To determine the effectiveness of the educational program based on self-regulation of learning in the level of knowledge about COVID-19 in the soldiers.

**Methods:**

Pre-experimental study with a pre-test and post-test design with a single group, carried out in 2020. 179 soldiers from Puno, Peru participated. Two expository-participatory sessions and a demonstrative workshop were developed. A valid (Aiken’s V = 0.69) and reliable (McDonald’s Omega = 0.81) questionnaire was used. The Friedman’s test was used to compare the results pre and post-test.

**Results:**

The level of knowledge about COVID-19 and in two of its dimensions changed from poor (pre-test) to regular (post-test). However, in the preventive measures dimension, it changed from poor (pre-test) to excellent (post-test).

**Conclusion:**

The proposed educational intervention was effective in increasing knowledge about COVID-19 in the soldiers, mainly in terms of preventive measures.

## RESUMEN

**Objetivo:**

Determinar la efectividad del Programa educativo basado en autorregulación del aprendizaje en el nivel de conocimiento sobre COVID-19 en militares.

**Métodos:**

Estudio preexperimental de diseño pretest y postest con un solo grupo, realizado en 2020. Participaron 179 militares de Puno, Perú. Se desarrollaron dos sesiones expositivo-participativas y un taller demostrativo. Se empleó un cuestionario válido (V de Aiken = 0,69) y confiable (Omega de McDonald = 0,81). Se utilizó el test de Friedman para comparar los resultados en pre y postest.

**Resultados:**

El nivel de conocimientos sobre COVID-19 y en dos de sus dimensiones cambió de deficiente (pretest) a regular (postest). Sin embargo, en la dimensión medidas preventivas, cambió de deficiente (pretest) a excelente (postest).

**Conclusión:**

La intervención educativa propuesta fue efectiva al incrementar los conocimientos sobre COVID-19 en militares, principalmente en cuanto a medidas preventivas.

## RESUMO

**Objetivo:**

determinar a efetividade do programa educacional baseado na autorregulação da aprendizagem a respeito do nível de conhecimento da COVID-19 em militares.

**Métodos:**

estudo pré-experimental de desenho pré-teste e pós-teste com um grupo só, realizado em 2020. Participaram 179 militares de Puno, Peru. Duas sessões expositivo-participativas e uma oficina demonstrativa foram desenvolvidas. Foi utilizado um questionário válido (V de Aiken = 0,69) e fiável (Ômega de McDonald = 0,81). O teste de Friedman foi empregado para comparar os resultados em pré e pós-teste.

**Resultados:**

o nível de conhecimento sobre a COVID-19 em duas das dimensões mudou de deficiente (pré-teste) para regular (pós-teste). Contudo, na dimensão de medidas preventivas, passou de deficiente (pré-teste) para excelente (pós-teste).

**Conclusão:**

a intervenção educacional proposta foi eficaz no aumento do conhecimento da COVID-19 entre os militares, principalmente em termos de medidas preventivas.

## INTRODUCTION

From the start of the pandemic to June 2022, approximately 6.3 million deaths from COVID-19^(1)^ were reported worldwide. More than 213,000 died from this viral disease in Peru^(2)^, country in which the number of deceased police officers exceeds that of any other occupational group^(3)^. From the beginning of this pandemic, the World Health Organization (WHO) recommended measures that contributed to reducing the mortality rate from this disease, such as mass testing, contact monitoring, isolation of cases, and social mobility restriction measures.

In this scenario, in Peru, military personnel were in charge of ensuring quarantine; in regions with scarce health personnel, such as the Puno region, they were in charge of verifying the use of masks, social distancing, and promoting the adoption of other COVID-19 prevention measures. However, recent research shows the negligent role of the armed forces, both government officials and military personnel, because they could have been vectors of the transmission of COVID-19^(4-5)^. Therefore, it is necessary to develop health interventions to promote the adoption of preventive measures in the among them.

Systematic reviews agree that the population has an adequate level of knowledge about COVID-19^(6-7)^. In the same line, two studies carried out in India agree that military personnel also have an adequate level of knowledge^(8-9)^. However, a study carried out in the Peruvian population indicates that more than half of those surveyed had little knowledge about the signs and symptoms of COVID-19^(10)^.

On the other hand, it has been reported that interventions that provide information lead to knowledge increment, adherence promotion and favors preventive measures against COVID-19^(11)^. In this regard, a study conducted in Israel reported that, through an educational intervention (short video), they were able to increase the knowledge score on COVID-19, the safety perception, and personal resilience^(12)^. In addition, it has been described that the mass media and the type of message the population receive influence the level of knowledge of public health and social change of behavior against the COVID-19 pandemic^(13)^.

Due to the impact of the pandemic on physical and psycho-emotional health, there is interest from the scientific community in improving knowledge, attitudes and practices about this disease through interventions that provide information and address concepts based on scientific evidence^(14)^, including strategies to raise awareness and use of education and communication technologies^(15)^. However, some interventions that include training only improve attitudes and practices, but not knowledge^(16)^.

From the sociocognitive perspective, the self-regulation based learning strategy consists of the deliberate organization of cognitive, behavioral, and environmental activities in which students establish goals that direct their learning and regulate their cognitions, motivations, and behaviors with the intention of achieving success in learning^(17)^. This model is widely used to improve knowledge and academic performance^(18)^; however, during the review of the literature on the subject, no programs based on self-regulation learning in the context of COVID-19 have been found.

As self-regulated learning models reinforces cognition by incorporating elements of the environment and context so that they can be incorporated into practice mainly through demonstration sessions or reinforcement workshops^(18)^ and complying with prevention measures for COVID-19 decreed by the Peruvian government, in coordination with the police authorities who provided ventilated spaces, a face-to-face educational intervention was carried out based on this model with the military forces in charge of guaranteeing compliance with preventive measures in the city of Puno during the health emergency.

## OBJECTIVE

To determine the effectiveness of the educational program based on self-regulated learning in the level of knowledge about COVID-19 among military forces.

## METHODS

### Ethical aspects

The study was approved by the Research Ethics Committee of the María Auxiliadora University. Likewise, the authorization of the Office of the Vice-rectorate for Research of the National University of the Altiplano of Puno and the General Commander of the IV Mountain Brigade was obtained. In compliance with Peruvian regulations, informed consent was given in person to military personnel, and the objective of the study, that participation is voluntary, and that answers will be stored anonymously, confidentially and only for research purposes was explained.

### Type of study

The study was pre-experimental, cross-sectional, with a preand post-test design for a Peruvian military personnel single group. For the report of this study, guidelines of the STROBE tool were followed.

### Setting and study period

This study was carried out between October and December 2020, when Peru was undergoing the first wave of COVID-19 infections^(19)^. Headquarters of the study were in the Manco Cápac barracks, located in the city of Puno. This department reports one of the highest rates of monetary poverty in Peru^(20)^. Regarding education, the illiteracy rate for people over 15 years of age is 8.7% and only 27.7% access higher education^(21)^.

### Population and sample: inclusion and exclusion criteria

The population consisted of 415 soldiers. The inclusion criteria were being a soldier in service and having the availability to participate in the educational sessions. Those who were on secondment, on study leave or had health problems that make it difficult for them to attend the sessions were excluded.

The sample consisted of 197 military personnel, calculated considering a confidence level of 95% and a margin of error of 5%. The selection of the participants was non-probabilistic but for convenience.

### Technique and instrument

A survey technique was used for data collection and a questionnaire as an instrument. The first section of the instrument inquired about demographic data such as age, sex, mother tongue and educational level; the second section evaluated the dependent variable of the study through questions with multiple alternatives about knowledge of COVID-19 such as: definition, transmission routes, incubation period, symptoms, preventive measures, and risk factors. These questions were prepared in accordance with the regulations issued by the Peruvian Ministry of Health and the recommendations of the WHO. It should be noted that some questions were adapted from previous research conducted in the Peruvian population^(22)^.

To determine the validity of the content, an expert assessment was carried out by a doctor and four nurses who are members of the multidisciplinary team of the COVID-19 in health establishments in Puno and who have extensive experience in the prevention of infectious diseases; they evaluated under three criteria: clarity, coherence and relevance. The scores obtained were quantified using Aiken’s V coefficient, which was 0.69, showing a positive evaluation of the scale.

Likewise, a pilot test was carried out in 18 (10% of the sample) soldiers from the Bolognesi barracks in the city of Juliaca. This police post presents similar socio-cultural and economic characteristics as the study headquarters. Through this activity, a question was modified, it was determined that participants take between 10 to 15 minutes to answer the survey, obtaining as a result a Kuder and Richardson coefficient of 0.839, indicating an adequate reliability.

### Analysis of construct validity and reliability of the instrument

The validity of the construct was evaluated through the exploratory factor analysis technique^(23)^, to analyze the representativeness of the items of the instrument. Following this, an average of 0.43 was obtained in the correlation matrix of the items that make up the instrument (with a value of p<0.05) and a value of 0.6 in the Kaiser Meyer Olkin Index (KMO). Finally, the Bartlett sphericity test was statistically significant (Chi2: 400.8; gl: 21; p<0.001)^(24)^, which demonstrates the susceptibility of the data through exploratory factor analysis. In addition, the same analysis showed that in its first matrix (communalities), representativeness of all the items within the factorial model (Table 1) and 2 factors showed an eigenvalue greater than 1, which explained more than 54% of the variance. The analysis of the factorial matrix revealed the representation of the items with a significant load to the found factor (Table 1).

**Table 1:** Communalities and Factorial Load of the items of the scale of knowledge about COVID-19 obtained in the exploratory factor analysis, 2020

Ítems	Communality	Factorial Load
P1	0.40	0.65
P2	0.47	0.73
P3	0.32	0.72
P4	0.37	0.74
P5	0.44	0.61
P6	0.48	0.64
P7	0.36	0.53
P8	0.34	0.65
P9	0.41	0.54
P10	0.37	0.75
P11	0.38	0.68
P12	0.28	0.64
P13	0.24	0.70
P14	0.31	0.69
P15	0.49	0.47
P16	0.46	0.63
P17	0.44	0.65
P18	0.35	0.74
P19	0.47	0.58
P20	0.38	0.69

**Table 2:** Characteristics of the study’s population, 2020

Characteristic	n (%)
Age (years)	
15-20	85 (47.5)
21-25	86 (48.0)
26-30	2 (1.1)
35-40	4 (2.2)
>40	2 (1.1)
Language	
Spanish	152 (84.9)
Quechua	4 (2.2)
Spanish and Quechua	23 (12.8)
Sex	
Male	161 (89.9)
Female	18 (10.1)
Degree of education	
Primary	1 (0.6)
Secondary	131 (73.2)
Technic	39 (21.8)
Higher	8 (4.5)

Additionally, with the data collected, the reliability coefficient was calculated (McDonald’s Omega index 0.71), indicating that the scale presents adequate internal consistency.

The knowledge scale consisted of 20 questions and 1 point was considered when the answer was correct and 0 when the answer was incorrect. A poor level of knowledge was considered when it had a score equal to or less than 10, a fair level from 11 to 15 points, a good level from 16 to 18, and an excellent level when it obtained a score greater than 18.

### Study protocol

The sequence of educational moments was fulfilled according to the stages of self-regulation of learning: planning, execution and self-reflection^(16)^; assumptions raised in self-regulated learning raised from the socio-cognitive approach to learning.

Regarding planning, the educational program was developed in the courtyard of the barracks, complying with the corresponding physical distancing and the proper use of personal protective equipment. A doctor with experience in managing COVID-19 patients, three nurses, and a head of military personnel participated. Three basic themes were programmed in nine different subgroups, at different times, with a duration of 60 to 100 minutes for each subgroup, the time being designated according to the theme and depending on the temporary availability of the soldiers.

Regarding the execution, the objectives of the investigation were explained to the participants in the first meeting and after having obtained the informed consent of each of them; in a following meeting, the pre-test was applied in order to assess the level of knowledge. Before developing each thematic area, they were assigned tasks such as a bibliographic review of the subject, exegetical technique with selected readings and questions that build the educational session.

The content of the program was as follows: Theme I: COVID 19, epidemiology, etiological agent, basic pathophysiology, transmission mechanisms, clinical picture and mention of the treatment delivered by the doctors. Theme II: Theoretic-practical workshop. Practical demonstrations and redemonstrations on the proper use of personal protective equipment, cough diagnosis, hand washing and antisepsis with alcohol gel were carried out, based on the recommendations of the Peruvian Ministry of Health. Theme III: Risk factors and complications derived from COVID-19.

Self-reflection was done after each learning session. This allowed military personnel to reinforce their knowledge and reflect on their mistakes. It should be noted that, to evaluate the effect of the educational program, the post-test was applied 20 days after the last educational session, using the same instrument. Finally, a triptych with a summary of the educational sessions was systematized in a participatory manner; this material was facilitated to the institution to be extended to other military headquarters in its jurisdiction.

Because the educational intervention was carried out with military troops which mostly come from marginal urban areas; difficulties in understanding the educational messages were manifested, requiring additional feedback sessions.

### Analysis of the results and statistics

Categorical variables were expressed in proportions. The results of the questionnaire were categorized as excellent, good, fair and deficient for each dimension and in the questionnaire in general. Categories were assigned according to the percentiles of the scores: “poor” (≤ 25th percentile), “fair” (> 25th percentile - 50th percentile), “good” (≥ 50th percentile-75th percentile) and “excellent”(> 75th percentile). The validation of the questionnaire was carried out with the McDonald Omega coefficient. The comparisons of the pre-test and post-test results by categories, in each dimension and in general, were made with the Friedman test. Values of p<0.05 were considered statistically significant. To perform the statistical analyses, the SPSS software, version 25.0 (IBM statistics), and Graphpad Prism 8.0 (GraphPad Software, San Diego, CA) were used.

## RESULTS

### Population characteristics

A total of 197 military service members were invited to participate and began the training; of which, only 179 attended all the educational sessions carried out (90.8% attendance). Characteristics of participants are presented in Table 2. When stratified by age, the range that groups the largest number of participants is 21-25 years (48%); most of the study participants spoke Spanish (84.9%), only 12.8% were bilingual (they speak Spanish and Quechua); in addition, the majority were males (89.9%) and 73% had secondary education.

### Evaluation of the effectiveness of the educational program by dimensions

**Figure 1:**
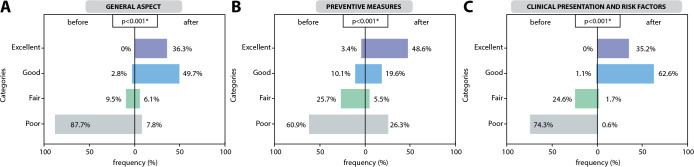
Evaluation of the effectiveness of the educational program by dimensions, 2020

*Note: Figure shows the results in the pre (before) and post (after) test. They were categorized as excellent, good, fair, and poor for each dimension. For the preand post-test comparisons, the Friedman test was used.*

Note: Figure shows the results of the pre (before) and post (after) test. The results were categorized as excellent, good, fair, and poor. *For the pre and post-test comparisons, Friedman test was used.

The 179 study participants performed preand post-tests. The results of the three dimensions of the instrument used are shown in Figure 1. In the results, in the first dimension, in General aspects, it can be observed that, in the pre-test, there was a higher frequency in the deficient category (87.7 %); After the intervention, the excellent (36.3%) and good (49.7%) categories resulted to appear more frequently in post-test, with a statistically significant difference (p<0.001) (Figure 1A). Then, in the dimension of Preventive measures, during the pre-test, the deficient category was obtained more frequently (60.9%); Meanwhile, when performing the post-test, the most frequent category was excellent; However, it was still possible to visualize that 26.3% obtained a poor result; these results were also significant (p<0.001) (Figure 1B). Finally, the evaluation of the dimension of *clinical presentation and risk factors* resulted in the same trend as the previous dimensions, being observed more a result in the poor category (74.3 %) in the pre-test; meanwhile, in the post-test, results between the categories excellent (35.2%) and good (62.6%), these results being also significant (p<0.001).

### General evaluation of the effectiveness of the educational program

**Figure 2:**
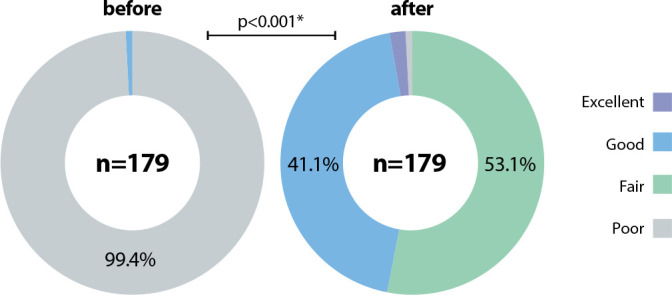
General evaluation of the effectiveness of the educational program, 2020

After evaluating the results by dimensions, shown in Figure 2, the overall results of the effectiveness of the educational program are as follows. Alarmingly, during the pre-test, 99.4% of participants scored in the poor category; after applying the educational program, post-test results were between fair (53.1%) and good (44.1%). Only 0.3% obtained a result in the poor category. As expected, these results were also significant (p<0.001).

## DISCUSSION

The main finding of this research was that the educational program based on the self-regulated learning model was effective, which was reflected in the military personnel’s increase in knowledge about COVID-19, from poor to fair and good levels. Moreover, regarding the analysis by dimensions, after the educational intervention, an excellent level of knowledge is reported in the Preventive Measures dimension, more than in the other dimensions evaluated.

This is attributed to the application of the self-regulated learning model, which involves cognitive and behavioral environmental activities^(18)^. Regarding cognitive activities, the military troops carried out focus and attention activities, and assigned tasks (bibliographic review of preventive measures for COVID-19), before the educational program. The most important explanation is that they took the responsibility of learning; additionally, this result is due to the demonstrations and redemonstrations of the practices of hand washing and correct use of the mask, with the message “by doing you learn”, a phrase that relates learning to doing, the environment and behaviors^(25)^. Another relevant aspect that has influenced the improvement of the knowledge of military personnel is that the self-assessment, which they carried out with a checklist, strengthened their knowledge; causing doctors and nurses, learning facilitators, to develop attitudes that correspond to metacognition and self-regulation.

Similar results were reported by interventions carried out in populations from different jurisdictions, in which education was used as a fundamental pillar. In this regard, in Israel, through an educational intervention, knowledge about COVID-19^(12)^ increased; in Cuba, approximately half of older adults, before the intervention, presented an inadequate level of general knowledge about COVID-19; while, after it, most presented adequate levels^(26)^. The similarity is attributed to the fact that both interventions were previously structured; while in Cuba the educational intervention was developed in three periods: a first moment to identify the learning needs, a second dedicated to the intervention and the last one aimed at evaluating the impact of the intervention on the study; the three moments were applied according to the phases of self-regulation of learning. When planning, the diagnosis was considered; during the execution, the development of face-to-face theoretical-practical sessions was considered; and during self-reflection, so it was the measurement of impact.

It should be noted that self-regulation of learning implied forecasting and delineation of an action plan; this was followed by the execution of the activation phase; finally, the self-reflection phase was rolled out, when the individual learns how to act. Currently, there are other conceptualizations that differ from the cyclical process of the three phases, but converge in that behavior associated with learning is goal-directed and controlled by feedback processes^(18,27)^.

Likewise, a study in Jordan, through messages, disseminated in the media, manages to promote behavior change against the virus disease with different methodologies; public media channels were important to increase awareness and social behavior change against the pandemic^(13)^. However, the learning sessions in the present study were theoretical-practical, applying self-regulation of learning. It is confirmed that, in order to structure the planning phase of an educational program, it is a priority to identify the strengths of the population as an active social actor, who not only receives knowledge, but also adds to the construction of learning as a self-regulating entity.

The deficient level of knowledge presented by the military personnel of Puno before the educational intervention could be due to gaps in access to education and health inequities as this department reports the highest poverty rates in Peru^(20-21)^. This finding is consistent with a study that reports a low level of knowledge about the signs and symptoms of COVID-19 in residents of Lima, Peru^(10)^. However, it disagrees with a research carried out in Jordan, in which they show that officers, superintendents and soldiers in service had a good level of knowledge about COVID-19^(28)^; also, with another study carried out in India, which highlights that the majority of soldiers have a high level of knowledge^(8)^. These differences could be attributed to several factors: the application of the instrument was face-to-face, where the young people responded without the option of accessing any information source; Added to this is the scant information regarding the prevention of COVID and that the majority of military personnel come from educational institutions with various limitations; reinforced, according to UNICEF, with the fact that there is inequity and inequality in the Peruvian educational system^(29)^.

Regarding the clinical picture and risk factors before the educational intervention, the results in the present were deficient in three quarters of the population. This result differs from a study in China, in which most of the participants correctly answered the most common clinical symptoms of COVID-19^(30)^. Meanwhile, in Lima, pregnant and women who had recently given birth from two Peruvian communities reveal a good level of knowledge about the prevention of COVID-19^(31)^. An explanation of these differences could be attributed to the fact that the patients received information from the health establishment, a different situation from the present study. It is reaffirmed that, in this part of the country, continuous educational programs for health promotion and disease prevention are lacking; likewise, epidemiological information from health professionals and those responsible for the situation of COVID 19 and its variants.

The implication of the study is that the findings show the effect of incorporating the model of self-regulation of learning in health education to increase knowledge of preventive measures against COVID-19, a key aspect at the beginning of the pandemic and which is becoming relevant at the end of the pandemic due to the appearance of variants of the SARS-COV2 virus due to vaccination requiring complementing these preventive measures^(32)^. In addition, through educational interventions, not only is compliance with prevention measures to prevent the transmission of diseases promoted in the population, but also an adequate scientific literacy is ensured^(33)^ giving instruments to the population to avoid other infectious diseases.

### Study limitations

The study has some limitations. Due to the type of study design, there is no way to fully attribute whether the pre-testing process actually influenced the results, thus external validity could be affected. Despite this, the instrument complies with all the assumptions of the construct, which could weigh the reliability of the results obtained in this study with its design. On the other hand, the participants who did not complete all the sessions due to duty responsibilities could present a different level of knowledge than the one reported. The type of non-probabilistic sampling used does not allow the results to be generalized to other populations; however, it could serve as a comparison with populations with similar characteristics. Finally, the pandemic context limited the development of a greater number of active dynamics in the educational sessions.

### Contribution of the study to the science of Nursing

The findings of the study reaffirm that, in the design of educational interventions aimed at young people, the use of participatory methodologies that emphasize the self-regulation of learning that uses the assumptions of the sociocognitive approach is effective in addressing cognitive, behavioral (being and doing) and socio-environmental processes. Aspect that is useful for Nursing professionals and students who during their preventive-promotional work develop training and training.

## CONCLUSIONS

The educational intervention based on self-regulation of learning developed with military troops was effective because the poor level of knowledge about COVID-19 that they presented in the pre-test changed to fair and good level of knowledge. However, in the dimension of Preventive measures, a greater change is reported, since it went from poor level (pre-test) to excellent level (post-test). Finally, it is recommended that, in educational sessions, the student is the protagonist of learning.
